# Rare Association of Dermatitis Herpetiformis and Chronic Plaque Psoriasis

**DOI:** 10.1002/ccr3.9669

**Published:** 2024-12-12

**Authors:** Fatima Ezzahraa Chekairi, Fouzia Hali, Zineb Chraibi, Wafaa Hliwa, Soumiya Chiheb

**Affiliations:** ^1^ Dermatology and Venereology Department Ibn Rochd University Hospital, Hassan II University of Casablanca Casablanca Morocco; ^2^ Gastroenterology Department Ibn Rochd University Hospital, Hassan II University of Casablanca Casablanca Morocco

**Keywords:** autoimmune diseases, celiac disease, dermatitis herpetiformis, plaque psoriasis

## Abstract

Dermatologists should consider investigating for coexisting autoimmune conditions such as dermatitis herpetiformis and/or coeliac disease, in patients with psoriasis presenting clinical signs and symptoms, in order to ensure an accurate diagnosis and improve patients' long‐term management.

## 1 Introduction

Dermatitis herpetiformis (DH) is a rare autoimmune and auto‐inflammatory bullous disease. It is characterized by a highly pruritic and symmetrical papulovesicular eruption, localized on the elbows, knees, back, and buttocks. The onset of DH typically occurs between the ages of 40 and 50 years. It shares some similar underlying factors with celiac disease (CD) and can be associated with gluten sensitivity [1].

On the other hand, plaque psoriasis is a chronic inflammatory skin disorder manifesting as erythematous and scaly plaques. According to the literature, psoriasis has been linked to autoimmune bullous diseases, in particular bullous pemphigoid, and may be associated with CD [2, 3].

However, to the best of our knowledge, the coexistence of dermatitis herpetiformis and plaque psoriasis has only been reported in a few isolated cases [4, 5]. We present a new case of DH development in a patient with chronic plaque psoriasis.

## 2 Case History/Examination

A 46 years old female patient with a history of chronic plaque psoriasis, was treated with topical corticosteroids, ultraviolet B (UVB) phototherapy, and injectable methotrexate (15 mg per week), with a good improvement of the skin lesions. However, following a relapse of her disease, the patient's plaque psoriasis was classified as moderate to severe on a psoriasis area and severity index (PASI) score of 14.6 and a dermatology life quality index (DLQI) of 15. Secukinumab was indicated, and a pre‐biological therapy screening was initiated.

**FIGURE 1:**
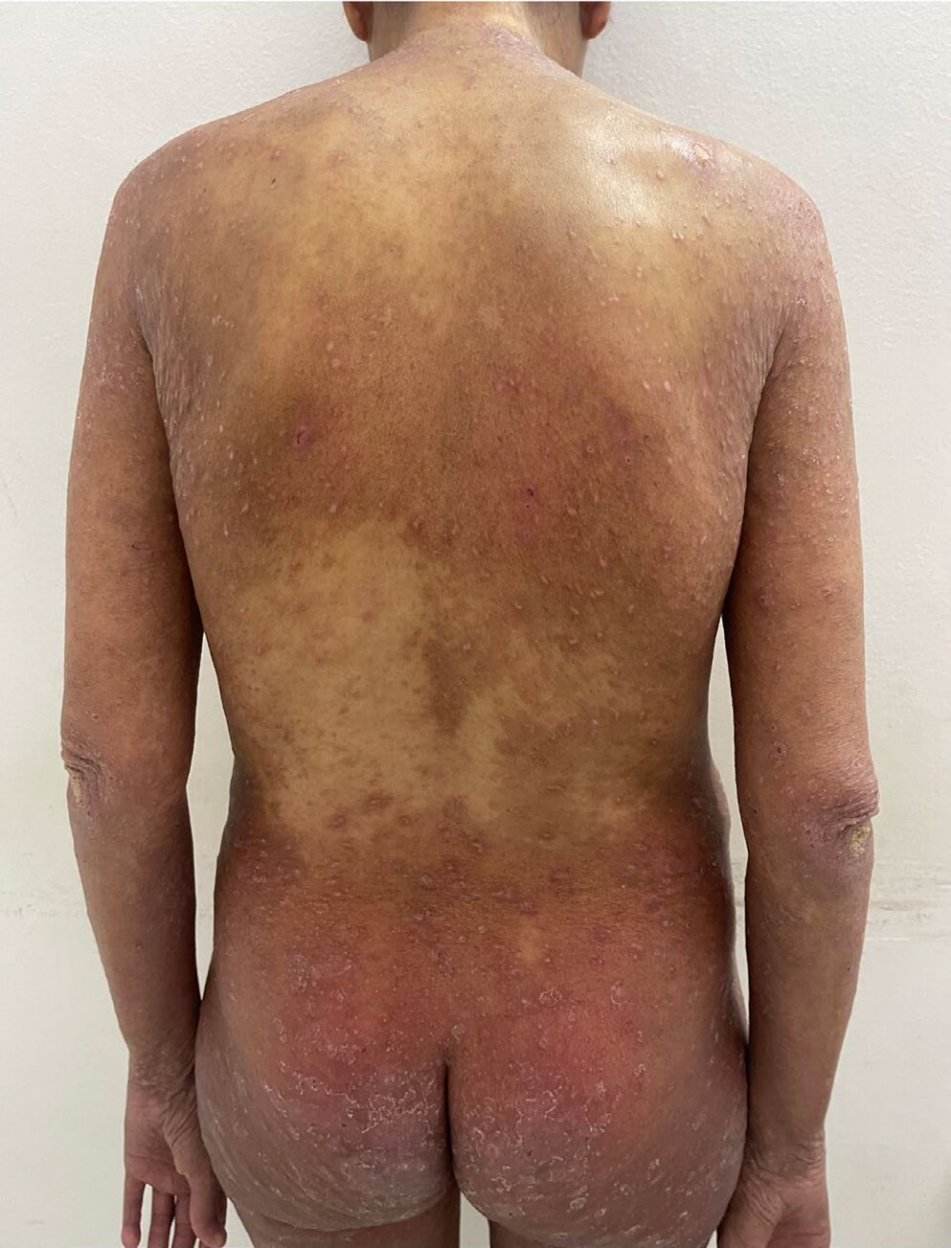
Dermatitis herpetiformis presenting as erythematous papules and herpetiform vesicles on the extensor surfaces of the upper limbs, buttocks, and back associated with erythematosquamous psoriatic plaques on elbows and buttocks.

Over the past 2 months, the patient developed intensely pruritic and erythematous papular lesions associated with herpetiform vesicles, on the extensor surfaces of her upper limbs, buttocks, and back. The lesions had a symmetrical distribution and were predominantly observed at the active edges of extensive psoriatic plaques, but also on healthy skin (Figure 1).

Moreover, the patient reported a recent onset of abdominal pain, bloating, and intestinal transit disorders, prompting a gastroenterology consultation.

## 3 Methods

Histological examination of a vesicular lesion revealed a parakeratotic skin tissue with an intraepidermal neutrophilic abscess containing eosinophils, and a dense junctional and papillary inflammatory infiltrate rich in neutrophils. The dermis was fibrous and contained a moderate inflammatory infiltrate of lymphocytes, histiocytes, and numerous neutrophils surrounding the vascular and adnexal sections. Direct immunofluorescence (DIF) revealed granular deposits of immunoglobulin A (IgA) in the dermal papillae, thereby confirming the diagnosis of dermatitis herpetiformis. Additionally, immunological tests demonstrated positive anti‐transglutaminase and anti‐gliadin antibodies, and a digestive endoscopy with duodenal biopsy confirmed the diagnosis of coeliac disease (CD). Treatment with dapsone at a daily dose of 50 mg combined to a gluten‐free diet was initiated.

## 4 Conclusion and Results

The treatment strategy for the concomitant DH and psoriasis in our patient resulted in a favorable improvement of both DH and psoriasis lesions. At 1 month follow‐up, a complete resolution of DH lesions and symptoms was observed.

## 5 Discussion

The coexistence of DH and psoriasis is a rare phenomenon. However, it presents a significant challenge for the accurate diagnosis and effective treatment of both autoimmune skin conditions, given their similarities. Indeed, the symptoms and clinical presentation of DH can be misdiagnosed as psoriasis flare‐ups, potentially resulting in delays in the appropriate management of both diseases. Additionally, they have a considerable impact on patients' quality of life, therefore, it is crucial for dermatologists to provide an early diagnosis and appropriate management.

A review of the literature suggests that DH and psoriasis share common underlying mechanisms. Patients with DH frequently exhibit elevated levels of interleukin‐8 (IL‐8), a cytokine that promotes the influx of neutrophils into the dermis and the formation of secondary vesiculo‐bullous lesions, as well as an increased expression of IL‐17 and IL‐36 correlated with the activity of the disease [6, 7]. In the case of psoriasis, IL‐36 and especially IL‐17 are implicated in its pathogenesis, and the hyperproliferation of keratinocytes may be responsible for excessive production of IL‐8 [8].

Furthermore, recent studies have indicated that psoriatic patients are at a higher risk of developing CD, particularly in case of severe psoriasis with an increased inflammatory activity, such as the case of our patient who was a candidate for a biological therapy [9, 10].

Moreover, a significant improvement of psoriasis symptoms has been documented in psoriatic patients with CD after a gluten‐free diet [4, 11]. This treatment approach should be considered in combination with topical corticosteroids, phototherapy, or other systemic treatments in patients with psoriasis presenting positive CD antibodies (IgA anti‐tissue transglutaminase, anti‐gliadin IgA and IgG, and IgA anti‐endomysial).

To the best of our knowledge, the coexistence of DH and plaque psoriasis has only been reported in a few isolated cases in the literature [4, 5].

Lee, Lobo, and Spelman [4] (2021) described a case of DH development in a 60 years old patient with chronic plaque psoriasis and CD. He presented multiple vesicles along the edges of psoriatic plaques on the back and hips, as well as vesiculo‐bullous lesions on his fingers. The patient was successfully treated with secukinumab for psoriasis, and a combination of dapsone and a gluten‐free diet for DH. Furthermore, the authors underscored the necessity of screening for CD and other autoimmune diseases such as DH, in patients with psoriasis.

Singh et al. [5] (2022) reported the coexistence of DH and chronic plaque psoriasis in a 12 years old girl, with no association to CD, suggesting an independent coexistence between DH and psoriasis. Moreover, they described the efficacy of a gluten‐free diet, methotrexate, and dapsone in managing the symptoms of both pathologies that were previously uncontrolled.

Similarly, our case report describes a rare association of DH with CD, and chronic plaque psoriasis with an unusual clinical presentation of pruritic papules and vesicles localized at the active edges of psoriatic plaques. This atypical manifestation suggests a Koebner's phenomenon that has been previously described in a small case series [12].

In addition, a combination therapy of dapsone and a gluten‐free diet successfully improved both DH and psoriasis lesions, resulting in a complete resolution of DH lesions and symptoms at 1 month follow‐up.

Overall, our case highlights the importance of identifying concomitant autoimmune conditions in patients with psoriasis, in particular DH associated with CD, and the therapeutic efficacy of a combination therapy on both DH and psoriasis symptoms. Furthermore, it underlines the necessity of a multidisciplinary approach involving dermatologists and gastroenterologists in order to ensure appropriate management, improve patients' quality of life and long‐term prognosis.

## Author Contributions

**Fatima Ezzahraa Chekairi:** conceptualization, data curation, writing – original draft. **Fouzia Hali:** supervision, validation. **Zineb Chraibi:** investigation. **Wafaa Hliwa:** investigation. **Soumiya Chiheb:** validation.

## Consent

A written and verbal consent was obtained from the patient before submission.

## Conflicts of Interest

The authors declare no conflicts of interest.

## Data Availability

The data supporting the findings of this study are available within the article. Additional source data are available from the corresponding author upon reasonable request.

## References

[1] C. N. Nguyen and S. J. Kim , “Dermatitis Herpetiformis: An Update on Diagnosis, Disease Monitoring, and Management,” Medicina (Kaunas, Lithuania) 57, no. 8 (2021): 843. 34441049 10.3390/medicina57080843 PMC8400185

[2] C. A. Maronese , N. Cassano , G. Genovese , C. Foti , G. A. Vena , and A. V. Marzano , “The Intriguing Links Between Psoriasis and Bullous Pemphigoid,” Journal of Clinical Medicine 12, no. 1 (2022): 328. 36615129 10.3390/jcm12010328 PMC9821109

[3] V. Vats , P. Makineni , S. Hemaida , et al., “Gluten Intolerance and Its Association With Skin Disorders: A Narrative Review,” Cureus 15, no. 9 (2023): e44549. 37790051 10.7759/cureus.44549 PMC10544948

[4] R. Lee , Y. Lobo , and L. Spelman , “Development of Dermatitis Herpetiformis in Chronic Plaque Psoriasis,” Case Reports in Dermatology 13, no. 1 (2021): 141–147. 33790758 10.1159/000512870 PMC7989782

[5] A. Singh , S. Ganguly , N. Chhabra , and V. Singh , “Rare Co‐Existent Dermatitis Herpetiformis and Psoriasis in a Child: A Causal Relationship?,” Cureus 14, no. 9 (2022): e29218. 36258965 10.7759/cureus.29218 PMC9569416

[6] K. H. Lee , A. Kronbichler , D. D. Park , et al., “Neutrophil extracellular traps (NETs) in autoimmune diseases: A comprehensive review,” Autoimmunity Reviews 16, no. 11 (2017): 1160–1173. 28899799 10.1016/j.autrev.2017.09.012

[7] A. Żebrowska , A. Woźniacka , K. Juczyńska , et al., “Correlation Between IL36α and IL17 and Activity of the Disease in Selected Autoimmune Blistering Diseases,” Mediators of Inflammation 2017 (2017): 8980534, 10.1155/2017/8980534. 28611508 PMC5458385

[8] S. Shimada , M. T. Mijiddorj , I. Kajihara , et al., “Increased Circulating Interleukin‐8 DNA Copies in Psoriasis,” Journal of Dermatology 51, no. 3 (2024): e70–e71, 10.1111/1346-8138.16988 Epub 2023 Oct 5. 37795802

[9] L. Li , L. Fu , L. Zhang , and Y. Feng , “Mendelian Randomization Study of the Genetic Interaction Between Psoriasis and Celiac Disease,” Scientific Reports 12, no. 1 (2022): 21508. 36513696 10.1038/s41598-022-25217-y PMC9747804

[10] A. Egeberg , C. E. M. Griffiths , L. Mallbris , G. H. Gislason , and L. Skov , “The Association Between Psoriasis and Coeliac Disease,” British Journal of Dermatology 177, no. 6 (2017): e329–e330. 28555725 10.1111/bjd.15684

[11] M. Passali , K. Josefsen , J. L. Frederiksen , and J. C. Antvorskov , “Current Evidence on the Efficacy of Gluten‐Free Diets in Multiple Sclerosis, Psoriasis, Type 1 Diabetes and Autoimmune Thyroid Diseases,” Nutrients 12, no. 8 (2020): 2316. 32752175 10.3390/nu12082316 PMC7468712

[12] G. Bonsmann and H. Hamm , “Köbner‐Phänomen Bei Dermatitis Herpetiformis Duhring [the Köbner Phenomenon in Duhring Dermatitis Herpetiformis],” Hautarzt 44, no. 1 (1993): 30–33. 8436505

